# Storage and disposal of expired medicines in home pharmacies: emerging public health problems

**DOI:** 10.31744/einstein_journal/2020AO5066

**Published:** 2020-02-13

**Authors:** Mayra Rodrigues Fernandes, Roberta Carvalho de Figueiredo, Luanna Gabriella Resende da Silva, Rafaela Silva Rocha, André Oliveira Baldoni

**Affiliations:** 1 Universidade Federal de São João del-Rei DivinópolisMG Brazil Universidade Federal de São João del-Rei, Divinópolis, MG, Brazil.

**Keywords:** Drug storage, Drug stability, Toxicology, Medical waste, Environment

## Abstract

**Objective:**

To characterize storage and disposal practices associated with expired medicines in home pharmacies of Primary Care users.

**Methods:**

Cross-sectional study based on data collected from 423 users of 15 Primary Care units located in a Brazilian city, between August 2014 and July 2016. Data were collected via face-to-face interviews. Categorical (demographic and socioeconomic characteristics) and continuous variables were expressed as proportions and means and standard deviations, respectively *.* Storage behaviors and disposal practices associated with unused and expired medicines were described as frequencies.

**Results:**

Most (83%) interviewees were female and approximately 70% had completed high school. The kitchen was the most common medicine storage place (58.6%). Approximately 75% of participants reported inappropriate medicine disposal practices.

**Conclusion:**

This study revealed high rates of inappropriate medicine disposal practices with direct impacts on pharmacological treatment and the environment. Continuing education of healthcare professionals and the general public is required to raise awareness about proper medicine use and disposal.

## INTRODUCTION

Medicines are the most common form of therapy in society and an integral part of almost all care levels.^1 , 2^ This contributes to ease of purchase, promoting self-medication and accumulation of medicines in houses. Medicines stored at home (“home pharmacy”) are often purchased over-the-counter, on recommendation from third parties and with no professional advice.^3^ Hence, medicine accumulation, storage of expired medicines and inappropriate disposal are common practices.^4^

Disposal of medicines within and beyond the expiry date is a public health concern. These products may cause health problems and generate emerging contaminants, and should therefore not have the same final destiny as common waste.^5 - 7^ Disposal in common waste or sewage networks may contaminate the soil, superficial waters ( *i.e* ., rivers, lakes and oceans) and groundwaters.^8^ Public administration, manufacturers, importers, distributers and sellers have a shared responsibility for product life cycles.^5 - 6^

Disposal and storage of expired products are not the only problems in home pharmacies. Storage conditions stand out as a significant factor in preservation and efficacy of medicines, as well as in prevention of domestic accidents.

Inadequate storage of medicines compromises quality, with potential harm to patients, the environment and even more to the aquatic microenvironment.^9^ Therefore, periodic inspection (at least twice a year) of medicines stored at home and disposal of expired or apparently damaged products is required to prevent poisoning or improper use.^10^ Pharmacists play a vital role in public education regarding proper storage and disposal of medicines.^11^

## OBJECTIVE

To identify and characterize storage and disposal practices associated with medicines stored at home among Primary Care users.

## METHODS

A cross-sectional study based on data from a survey of individuals aged 18 years or older seen at Primary Care Units, located in the city of Divinópolis (State of Minas Gerais/MG). Population of Divinópolis was estimated at 230,848 inhabitants at the time of this study.^12^ The city comprises 34 Primary Care Units and five public pharmacies belonging to the Medicines of the Basic Component of Pharmaceutical Services.

A standardized questionnaire was developed based on literature data and study objectives and submitted to appreciation by three pharmacoepidemiology experts for correction and adaptation. The instrument was then tested in a pilot study with ten participants to check for question understanding. Pilot study participants were not included in this sample.

Briefly, the questionnaire aimed to characterize home pharmacies. The sample comprised 423 Primary Care Units users. The following sample calculations were employed: 50% prevalence, given the lack of scientific evidence regarding the prevalence of expired medicines in home pharmacies, 5% precision, 95% confidence level and 10% of losses. Fifteen primary care units were randomly selected for this study. Units located in rural areas and in places with high levels of urban violence were not included.

Individuals were invited to participate in the study prior to or after medical visits in selected units. The interviewer explained the study objectives and collected patient contact information. Data were collected between August 2014 and July 2016 via face-to-face interviews. Interviews were conducted by trained interviewers using a structured questionnaire including questions about medication use, storage, expiry date and disposal. Sociodemographic data were also collected.

The questionnaire allowed characterizing home inventories, storage and disposal practices, and collection of data about exposure to humidity and/or high temperatures or direct sunlight. Use of raised storage areas (1.5m high or higher) out of children’s reach was also interrogated.

Inappropriate storage was defined as exposure to direct sunlight, humidity and/or dirt, or within reach of children (less than 1.5m high). Storage of thermolabile products outside the fridge, in the fridge door or freezer was defined as inappropriate storage, whereas storage away from fridge walls, in the middle shelf, lower portion or vegetable drawer was defined as appropriate storage, as long as medicines were kept in their original packaging or, in the case of insulin, in sealed plastic or metal containers.^13^

Inadequate disposal was defined as disposal in domestic waste, toilet, bathroom/kitchen sink and rivers/lakes. Burying and/or storage of expired medicines or donation to neighbors or relatives were also defined as inadequate practices. Adequate disposal was defined as drop off at care units, public or private pharmacies.

Categorical variables (demographic and socioeconomic characteristics) were expressed as proportions. Storage and expired medicine disposal practices were expressed as frequencies. Data were entered into Epi Info™, version 7. Statistical analyses were conducted using software (Stata, version 12.0, Stata Corporation, College Station *,* United States).

### Ethical aspects

This study was approved by the Research Ethics Committee of *Universidade Federal de São João del-Rei* (UFSJ), Campus Centro-Oeste Dona Lindu, CAAE: 3091.231.4.0.0.5545, opinion 655.930.

## RESULTS

A total of 612 individuals seen at 15 Primary Care Units were invited to participate in this study. Of these, 26.6% (n=163) refused to participate and 4.3% (n=26) agreed to participate then chose to drop out. The final sample comprised 423 interviews. Sample distribution was as follows: 83% of participants were women (n=351), 70% had completed high school and 4% higher education. The most common medicine storage place was the kitchen, followed by the bedroom and the living room (58.6%, 57.2%, and 14.4% respectively), as shown in table 1 .

**Table 1:** Places and conditions of medicine storage in home pharmacies reported by Primary Care users

	n (%)
Place of storage	
Kitchen	248 (58.6)
Bedroom	242 (57.2)
Living/pantry room	61 (14.4)
Bathroom	9 (2.1)
Others*	6 (1.4)
Total^†^	566
Storage conditions	
Within reach of children	281 (66)
Exposure to sunlight	53 (13)
Thermolabile products at inappropriate places^‡^	32 (8)
Exposure to humidity	17 (4)
Total	383

* Others: bag, balcony and corridor; ^†^ more than one answer per interviewee accepted; ^‡^ thermolabile products at inappropriate places: outside the fridge, in fridge door and/or freezer.

Approximately 8% (n=32) of interviewees reported inappropriate storage of thermolabile products and 17% reported exposure to sunlight and humidity. Storage within reach of children was often reported (66%).

Disposal of expired medicines in inappropriate places was reported 437 times (disposal in domestic waste reported 257 times). Storage and/or donation of expired medicines was reported by 1.5% (n=8) of participants. Appropriate disposal of expired medicines (drop off at primary care units or public/private pharmacies) was reported by 8.3% (n=46) of interviewees. A similar number of interviewees (8.0%) reported having received professional advice regarding disposal. Inappropriate disposal of expired medicines was reported by 75.4% of participants. Disposal directly in the environment ( *i.e.* , domestic waste, toilet, kitchen/bathroom sink, rivers and lakes) accounted for 74% of these cases ( Table 2 ).

**Table 2:** Disposal practices associated with expired medicines stored in the homes of Primary Care users

Disposal practices	n (%)
Correct practices	
Drops off at primary care unit or gives back to health agent	33 (6.0)
Drops off at public/private pharmacy	13 (2.3)
Incorrect practice	
Stores for future use	7 (1.3)
Donates to neighbors/friends/relatives	1 (0.2)
Disposes in domestic waste	257 (46.7)
Disposes in toilet	31 (21.8)
Disposes in kitchen/bathroom sink, rivers or lakes	52 (9.5)
Others*	67 (12.2)
Total	550 (100)

* Others: burns, buries or uses until there is none left. More than one answer per interviewee accepted.

## DISCUSSION

High prevalence of women at Primary Care Units has been reported in a study by Malta et al., In that study, health-related concerns were more frequently expressed by female compared to male patients.^14^

The kitchen was the most common place of medicine storage in this sample. Ease of access, proximity to water filters or other liquids suitable for medication intake and easy access to kitchenware, such as spoons for dosing of solutions and suspensions, may explain this finding.^15^ The fact that storage in humid ( *e.g.* , the bathroom), hot or cold places may affect physical and chemical properties of drugs and interfere with their efficacy effectiveness should be emphasized. Presence of sanitation and chemical products in bathrooms may also increase the chances of contamination.^16^ Similar findings have been reported by Schwingel et al.,^3^ who described 59% of participants storing medicines in the kitchen, 30% in the bedroom and 14.0% in the living room. Storage of medicines within reach of children, the leading cause of human poisoning in Brazil, is another important issue that needs to be addressed.^17^ According to the National System of Toxicologic and Pharmacological Information [ *Sistema Nacional de Informações Tóxico Farmacológicas* ] (SINITOX) estimates,^18^ 22,395 cases of drug poisoning were reported between 2003 and 2012, with 17,725 hospital admissions and 75 deaths in children aged under 5 years.^17 , 19^

Storage of thermolabile products also require special attention, given the direct impact on therapeutic effects.^20^ Use of inappropriately stored and/or expired medicines may cause serious health problems, such as poisoning and severe adverse events. The expiry date is the time period during which maximal therapeutic effects with minimal adverse events and retention of physical, chemical and pharmacological properties can be expected, provided the product is stored according to manufacturer’s recommendations.^21 , 22^ The National Health Surveillance Agency [ *Agência Nacional de Vigilância Sanitária* ] (ANVISA) recommends disposal of unused medications. Should medications purchased over-the-counter be stored at home for emergency situations, the expiry date should be constantly checked, and expired or unused products disposed.^23^ Abovementioned problems may be mitigated or solved with proper pharmaceutical advice regarding medication storage or by reading of package inserts.^24^

Inadequate disposal of expired medicines is also a significant public health and environmental concern in the United States. Frequent disposal in domestic waste, sinks and toilets in that country^25^ has been associated with increased concentration of drugs in wastewater and effluents.^26 , 27^ Low rates of appropriate disposal reflect lack of education among health professionals, as pointed out by Costa, in a study conducted in Campina Grande (State of Paraiba, Brazil). In that study, only a small proportion of participants reported having received professional advice about drug disposal.^28^

The legislation is deficient and directed to health facilities, not informing details that guide the population^6^ Thus, there is rarely adequate collection of this waste by public and private health institutions.^29^ However, it is clear that this policy was not effectively implemented, as there is a lack of disclosure to the population about the collection. In addition, reverse logistics involves financial resources that companies are not always willing to fund.

Therapeutic waste collection programs reduce the amount of medicines reaching the environment. As professionals involved in all types of medication-related activities, pharmacists should take responsibility for the final stage of drug life cycle and be concerned about patient safety and the environment.^30^ Pharmacists should also use related knowledge to encourage and promote reflection and debate among health professionals, managers, politicians and the public at large, in an effort to mitigate the impacts of inappropriate medicine disposal and improve health and quality of life.^11^

The general public should be co-responsible for this process, properly informed and involved in awareness raising about waste generation and rational use of medicines, as well as about measures required to reduce surplus medicine accumulation due to unnecessary purchases or lack of compliance with therapeutic regimens prescribed.^31^

This sample comprised exclusively users of the National Public Health System (SUS - *Sistema Único de Saúde* ). Therefore, findings cannot be extrapolated to users of the private health system. However, methodological rigor should be emphasized. Diversity sampling and accurate responses obtained via home visits to all participants allowed true identification and characterization of medicine storage and disposal practices among primary care users.

## CONCLUSION

The kitchen was the most common place of medicine storage. Most interviewees reported inappropriate medicine storage behaviors, such as storage at inadequate temperatures, exposure to light, humidity and dust and/or storage within reach of children. High rates of inappropriate disposal practices and lack of related information emphasize the need for continuing education of health professionals and the public at large for increased awareness about proper medication use and disposal. More strict surveillance to ensure compliance with national and state laws regulating pharmaceutical reverse logistics are also required to mitigate potential clinical and environmental impacts of inadequate medicine disposal.
