# Supplementary material for: Storage and disposal of expired medicines in home pharmacies: emerging public health problems
Source: Einstein (Sao Paulo). 2020 Feb 13;18:eAO5066. doi: 10.31744/einstein_journal/2020AO5066 (PMC7018094; doi:10.31744/einstein_journal/2020AO5066)
Supplement: Translation

# Translation: Armazenamento e descarte dos medicamentos vencidos em farmácias caseiras: problemas emergentes para a saúde pública

**Authors:** Mayra Rodrigues Fernandes, Roberta Carvalho de Figueiredo, Luanna Gabriella Resende da Silva, Rafaela Silva Rocha, André Oliveira Baldoni

**Affiliations:** 1 Brasil Universidade Federal de São João del-Rei, Divinópolis, MG, Brasil.

**Keywords:** Armazenamento de medicamentos, Estabilidade de medicamentos, Toxicologia, Resíduos de serviços de saúde, Meio ambiente

## Abstract

**Objetivo:**

Caracterizar o armazenamento e o descarte de medicamentos vencidos contidos em farmácias caseiras de usuários da Atenção Primária à Saúde.

**Métodos:**

Estudo transversal, realizado com 423 usuários de 15 unidades de saúde da Atenção Primária em um município brasileiro. Os dados foram coletados de agosto de 2014 a julho de 2016, por meio de entrevistas face a face. As características demográficas e socioeconômicas foram descritas por meio de proporções para as variáveis categóricas. As formas de armazenamento e o descarte de medicamentos vencidos ou não vencidos foram descritos em forma de frequência.

**Resultados:**

Dentre os entrevistados, 83% eram do sexo feminino e aproximadamente 70% possuíam Ensino Médio completo. A cozinha foi o local mais citado para armazenamento de medicamentos (58,6%). Cerca de 75% dos participantes relataram descartar os medicamentos de forma incorreta.

**Conclusão:**

O estudo evidenciou que grande proporção dos entrevistados possui hábitos incorretos de descarte, que, por sua vez, impactam diretamente no tratamento medicamentoso e na natureza. Assim, é necessária a educação continuada dos profissionais de saúde e da população, a fim de conscientizar a população sobre a correta utilização e o descarte de medicamentos.

## INTRODUÇÃO

Os medicamentos encontram-se inseridos em praticamente todas as esferas de atenção à saúde, porque são considerados a forma mais comum de terapia na sociedade.^1 , 2^ Isso aumenta a facilidade na aquisição de medicamentos, levando à prática de automedicação e ao consequente acúmulo dessas substâncias nas residências. O medicamento estocado em domicílio, também chamado de “farmácia caseira”, é adquirido, muitas vezes, por conta própria, por indicação de terceiros e sem orientação de um profissional.^3^ Assim, o acúmulo de medicamentos, a expiração do prazo de validade e o descarte inapropriado dessas substâncias tornam-se práticas comuns em nosso meio.^4^

O descarte de medicamentos, vencidos ou não, é uma preocupação para a saúde pública, visto que podem ocasionar danos à saúde e gerar contaminantes emergentes. Por isso, eles não devem ter a mesma destinação final de resíduos comuns.^5 - 7^ O descarte no lixo comum ou na rede de esgoto pode contaminar o solo; as águas superficiais, como rios, lagos e oceanos; e águas subterrâneas, como os lençóis freáticos.^8^ O poder público, os fabricantes, os importadores, os distribuidores e os comerciantes têm responsabilidade compartilhada pelo ciclo de vida do produto.^5 - 6^

Todavia, o vencimento e o descarte não são os únicos problemas encontrados na farmácia caseira. O armazenamento correto surge como fator importante para a conservação e a efetividade do medicamento, assim como para a prevenção de acidentes domésticos.

Medicamentos, quando mal armazenados, têm sua qualidade comprometida, causando danos ao paciente e ao meio ambiente, sobretudo ao microambiente aquático.^9^ Assim, fazem-se necessárias inspeções periódicas dos medicamentos que compõem a farmácia caseira, pelo menos duas vezes ao ano, para que sejam descartados os vencidos e os que estão com qualidade aparentemente comprometida, evitando intoxicações e possíveis usos equivocados.^10^ Deste modo, o farmacêutico tem fundamental importância na orientação da população sobre a forma correta de uso, armazenamento e descarte desses insumos terapêuticos.^11^

## OBJETIVO

Identificar e caracterizar o armazenamento e o descarte de medicamentos contidos em farmácias caseiras de usuários da Atenção Primária à Saúde.

## MÉTODOS

Trata-se de um estudo transversal, que utilizou dados de um inquérito realizado com indivíduos com 18 anos ou mais, atendidos em unidades de Atenção Primária à Saúde do município de Divinópolis (MG), cuja população estimada consistia em 230.848 habitantes no período em que o estudo foi realizado.^12^ O município possui 34 centros de Atenção Primária à Saúde e cinco farmácias públicas do Componente Básico da Assistência Farmacêutica.

Foi utilizado um questionário padronizado elaborado com base na literatura e no objetivo do estudo e que, posteriormente, foi analisado por três *expertises* pesquisadores da área de farmacoepidemiologia, para possíveis alterações e adequações. Após esta fase, o instrumento foi testado em um estudo piloto com dez participantes, para análise da compreensão das perguntas. Os participantes do estudo piloto não foram incluídos na pesquisa.

Em síntese, esse questionário visava à caracterização das farmácias caseiras, e sua amostra foi de 423 usuários atendidos na Atenção Primária à Saúde, levando-se em conta o seguinte cálculo amostral: prevalência de 50%, dada a escassez de evidências científicas sobre prevalência de medicamentos vencidos em domicílios, precisão de 5%, nível confiança de 95% e 10% de perdas. Foram sorteadas 15 unidades de saúde para realização da pesquisa; aquelas localizadas na zona rural e em locais de elevada taxa de violência urbana foram excluídas do sorteio.

Os indivíduos foram convidados a participar do estudo quando estavam nas unidades de saúde sorteadas, antes ou após o atendimento, onde o entrevistador explicou o objetivo do estudo e pegou os dados dos pacientes para um contato posterior. Esses dados foram coletados no período de agosto de 2014 a julho de 2016, por meio de entrevistas face a face, por entrevistadores treinados, utilizando-se um questionário estruturado, que abordou questões sobre uso, armazenamento, prazos de validade e descarte dos medicamentos, além de características sociodemográficas dos participantes.

Por meio do questionário, foi possível fazer a caracterização dos estoques domiciliares, além da identificação da forma de armazenamento e descarte dos medicamentos e, ainda, se eram expostos à umidade e/ou a temperaturas elevadas ou ficavam sob incidência direta de luz, bem como dos medicamentos armazenados em locais com altura mínima de 1,5m (fora do alcance de crianças).

Foi considerado inadequado o armazenamento de medicamentos expostos diretamente à luz solar ou à umidade e/ou sujeira e medicamentos armazenados sob alcance de crianças (armazenamento com altura inferior a 1,5m). Termolábeis fora da geladeira, em sua porta ou dentro do congelador foram considerados como em formas inadequadas de armazenamento, enquanto os termolábeis armazenados nas prateleiras do meio, na parte inferior ou na gaveta de verduras, longe das paredes, em sua embalagem original e acondicionados, no caso de frascos de insulinas, em recipiente plástico ou de metal, com tampa, foram considerados como em condutas corretas para armazenamento.^13^

Descartes incorretos de medicamentos foram aqueles realizados em lixo doméstico, vaso sanitário, pia do banheiro/cozinha e em rios/lagos. Enterrar e/ou guardar medicamentos vencidos, assim como doá-los a vizinhos ou parentes, também foram considerados comportamentos inadequados. Já o descarte correto foi aquele em que os usuários relataram devolver os medicamentos em unidades de saúde, farmácias públicas ou privadas.

As características demográficas e socioeconômicas dos entrevistados foram descritas por meio de proporções para as variáveis categóricas. As formas de armazenamento dos medicamentos foram descritas por meio de frequências, assim como o descarte dos vencidos. Os dados foram inseridos no Epi Info™, versão 7, e as análises foram realizadas no software Stata, versão 12.0 ( *Stata Corporation, College Station,* Estados Unidos).

### Aspectos éticos

O estudo foi aprovado pelo Comitê de Ética em Pesquisa da Universidade Federal de São João del-Rei (UFSJ), *Campus* Centro-Oeste Dona Lindu, CAAE: 3091.231.4.0.0.5545, parecer 655.930.

## RESULTADOS

Foram abordadas 612 pessoas atendidas em 15 unidades de saúde, das quais 26,6% (n=163) recusaram participar do estudo e 4,3% (n=26) desistiram, totalizando 423 entrevistas realizadas. Dos entrevistados, 83% (n=351) eram do sexo feminino. Quanto à escolaridade, 70% possuíam Ensino Médio completo e 4% cursaram o Ensino Superior. Quanto ao armazenamento de medicamentos, os locais mais citados foram cozinha (58,6%), seguida do quarto (57,2%) e sala (14,4%), como demonstra a tabela 1 .

**Tabela 1:** Locais e condições de armazenamento dos medicamentos contidos em farmácias caseiras de usuários da Atenção Primária à Saúde (n=423)

	n (%)
Local de armazenamento	
Cozinha	248 (58,6)
Quarto	242 (57,2)
Sala/copa	61 (14,4)
Banheiro	9 (2,1)
Outros*	6 (1,4)
Total^†^	566
Condições de armazenamento	
Armazenados ao alcance de crianças	281 (66)
Expostos à luz solar	53 (13)
Termolábeis em lugares inapropriados^‡^	32 (8)
Expostos à umidade	17 (4)
Total	383

* Outros: bolsa, varanda e corredor;^†^admitiu-se mais de uma resposta por entrevistado;^‡^termolábeis em lugares inapropriados: fora da geladeira, porta da geladeira e/ou congelador.

Aproximadamente 8% (n=32) dos entrevistados armazenavam incorretamente os termolábeis. Em relação à exposição à luz e à umidade, 17% dos entrevistados guardavam os medicamentos sob tais condições. Elevada proporção (66%) relatou armazenar medicamentos ao alcance de crianças.

O descarte de medicamentos vencidos em locais considerados inapropriados foi relatado 437 vezes, dos quais 257 foram no lixo doméstico. Ainda sobre os resultados encontrados no estudo, 1,5% (n=8) dos participantes disseram ter o hábito de guardar e/ou doar medicamento com data de validade expirada. Das pessoas entrevistadas, 8,3% (n=46) relataram fazer o descarte de vencidos corretamente, devolvendo às farmácias públicas ou privadas ou entregando à unidade de saúde, resultado aproximado daqueles que relataram receber orientação profissional quanto ao descarte (8,0%). Observou-se que 75,4% dos pacientes relataram descartar inadequadamente os medicamentos vencidos, sendo que 74% realizavam o descarte diretamente no meio ambiente, como lixo doméstico, vaso sanitário, pias da cozinha e/ou banheiro, rios e lagos ( Tabela 2 ).

**Tabela 2:** Hábitos de descarte de medicamentos vencidos das farmácias caseiras de usuários da Atenção Primária à Saúde

Hábitos de descartes	n (%)
Hábitos corretos	
Devolve à unidade ou ao agente de saúde	33 (6,0)
Devolve à farmácia pública/privada	13 (2,3)
Hábitos incorretos	
Guarda para usar outra vez	7 (1,3)
Doa aos vizinhos/amigos/parentes	1 (0,2)
Joga no lixo doméstico	257 (46,7)
Joga no vaso sanitário	31 (21,8)
Descarta na pia do banheiro, pia da cozinha, em rios ou lagos	52 (9,5)
Outros*	67 (12,2)
Total	550 (100)

* Outros: queima, enterra ou usa até acabar. Admitiu-se mais de uma resposta por entrevistado.

## DISCUSSÃO

A alta prevalência de mulheres encontradas nas unidades de saúde também foi encontrada em um estudo conduzido por Malta et al., no qual pacientes do sexo feminino relataram, com maior frequência, preocupação com a saúde em relação aos homens.^14^

Quando questionados sobre os locais de armazenamento dos medicamentos em suas residências, a cozinha foi citada pela maioria dos participantes, possivelmente pela acessibilidade do local, por geralmente estar perto de filtros de água e ou na presença de outros líquidos que podem ser ingeridos com o medicamento e de utensílios, como colheres para medida de soluções e suspensões.^15^ É importante lembrar que armazená-los em lugares úmidos, como o banheiro, ou em locais quentes ou frios, pode alterar as propriedades físico-químicas dos medicamentos, interferindo em sua efetividade, além de ser um ambiente onde há saneantes e produtos químicos, aumentando a chance de contaminação.^16^ Esses resultados vão ao encontro do estudo guiado por Schwingel et al.,^3^ no qual 59% dos participantes armazenavam os medicamentos na cozinha, 30% no quarto e 14,0% na sala. Outro ponto importante a ser debatido é o armazenamento de medicamentos ao alcance de crianças, visto que é o principal agente causador de intoxicação em seres humanos no Brasil.^17^ Segundo dados do Sistema Nacional de Informações Tóxico Farmacológicas (SINITOX),^18^ no período de 2003 a 2012, foram identificados, 22.395 casos de intoxicações, sendo que 17.725 resultaram em hospitalizações e 75 em óbitos, somente em crianças com idade inferior a 5 anos.^17 , 19^

Além disso, os termolábeis merecem atenção especial quanto ao armazenamento, visto que seu acondicionamento interfere diretamente nos resultados terapêuticos.^20^ O uso de medicamentos mal acondicionados e/ou vencidos acarreta sérios danos à saúde, como intoxicações e eventos adversos mais acentuados, uma vez que o prazo de validade é o espaço de tempo em que o fármaco exerce o máximo de sua ação terapêutica e o mínimo de reações adversas, conservando as características químicas, físicas e farmacológicas, quando estocado de acordo com as orientações do fabricante.^21 , 22^ A Agência Nacional de Vigilância Sanitária (ANVISA) orienta a não armazenar sobras de medicamentos em casa. Quando é necessário ter em casa alguns medicamentos de venda isenta de prescrição médica para alguma emergência, deve-se checar a data de validade constantemente, para retirada dos que apresentem a validade expirada ou estão há muito tempo fora de uso.^23^ Os problemas supracitados podem ser reduzidos ou solucionados se houver orientação farmacêutica quanto aos locais de armazenamento, ou se realizada a leitura das especificações contidas nas bulas.^24^

Em relação ao descarte de fármacos vencidos, nos Estados Unidos, a prática inadequada também é um grande problema ambiental e de saúde pública. No referido país, as principais formas de descarte de medicamentos também são lixos domésticos, pias e vaso sanitário,^25^ fato que aumenta a concentração de fármacos em águas residuais tratadas e efluentes de recepção de água.^26 , 27^ A baixa proporção de descarte correto está associada a pouca orientação recebida pelos profissionais de saúde, observação também encontrada no estudo realizado por Costa, em Campina Grande (PB), no qual uma baixa proporção dos participantes da pesquisa relatou receber algum tipo de orientação profissional quanto ao descarte.^28^

A legislação é deficiente e direcionada para estabelecimentos de saúde, não informando detalhes que orientem a população.^6^ Assim, raramente existe coleta adequada desses resíduos por parte dos estabelecimentos públicos e privados de saúde.^29^ No entanto, percebe-se que essa política não foi efetivamente implementada, pois falta divulgação para a população sobre a coleta. Além disso, para realizar a logística reversa envolvem-se recursos financeiros, nos quais as empresas nem sempre estão dispostas a custear.

Os programas de coleta desses insumos terapêuticos reduzem a quantidade de medicamentos que chegam ao meio ambiente. O farmacêutico, como profissional envolvido em todas as ações relacionadas ao medicamento, deve incluir em suas responsabilidades a preocupação com o final do ciclo de vida dele, buscando tanto a segurança do paciente, como do meio ambiente.^30^ Além disso, com base nos conhecimentos relacionados ao tema, o farmacêutico deve incentivar e promover a reflexão e a discussão acerca do assunto envolvendo profissionais de saúde, gestores, políticos e a população, com o intuito de minimizar os efeitos do descarte inadequado de medicamentos e propiciar melhora na saúde e na qualidade de vida de uma população.^11^

A população também deve ser corresponsável desse processo, devendo estar esclarecida e envolvida no processo de conscientização da geração de resíduos e do uso racional de medicamentos, bem como as medidas necessárias para diminuir as sobras decorrentes de aquisição desnecessária ou do não cumprimento do esquema terapêutico proposto.^31^

Em relação às limitações do estudo, é importante ressaltar que o trabalho foi realizado apenas com usuários do Sistema Único de Saúde (SUS), e os resultados não podem ser extrapolados para usuários do sistema privado de saúde. Por outro lado, vale destacar o rigor metodológico do estudo para que se conseguissem a diversidade amostral necessária e a acurácia das respostas frente a realização das visitas domiciliares com todos os participantes, para que fosse possível a real identificação e a caracterização do armazenamento e do perfil de descarte de medicamentos contidos em farmácias caseiras de usuários da Atenção Primária à Saúde.

## CONCLUSÃO

A cozinha foi o lugar mais frequentemente utilizado para armazenar medicamentos, e a maioria dos entrevistados acondicionava medicamentos em temperaturas inadequadas, expostos à luz, umidade, poeira e/ou alcance de crianças. A elevada frequência de descarte inadequado, juntamente da escassez de informação sobre o assunto, reforça a real necessidade de uma educação permanente dos profissionais de saúde e da população em geral, para a conscientização da correta utilização e descarte de medicamentos. Ademais, são necessárias ações mais rígidas para fiscalizar o cumprimento de leis nacionais e estaduais relacionadas à logística reversa dos medicamentos, de forma a minimizar os potenciais impactos clínicos e ambientais causados pelo descarte incorreto de medicamentos.
